# A double SORLIP1 element is required for high light induction of *ELIP* genes in *Arabidopsis thaliana*

**DOI:** 10.1007/s11103-013-0130-4

**Published:** 2013-09-27

**Authors:** Ana M. Rus Alvarez-Canterbury, Daisy Janette Flores, Keykhosrow Keymanesh, Kevin To, Judy Ann Brusslan

**Affiliations:** 1 Department of Biological Sciences, California State University, Long Beach, 1250 Bellflower Blvd., Long Beach, CA 90840-9502 USA; 2 Present Address: Norris Comprehensive Cancer Center, University of Southern California, 1441 Eastlake Ave., Los Angeles, CA 90033 USA

**Keywords:** ELIP, SORLIP, High light signaling, Lhca2

## Abstract

Promoter elements that contribute to high light (HL) induction of the Arabidopsis *ELIP1* gene were defined using a transgenic promoter-reporter system. Two adjacent SORLIP1 elements (double SORLIP1, dSL) were found to be essential for HL induction of a GUS reporter gene. The dSL element was also found to be essential for HL induction conferred by the *ELIP2* promoter. SORLIP1 elements were enriched in *ELIP* promoters throughout the plant kingdom, and showed a clade-specific pattern of gain or loss that suggested functionality. In addition, two G-box elements were found to redundantly contribute to HL induction conferred by the *ELIP1* promoter.

**Electronic supplementary material:**

The online version of this article (doi:10.1007/s11103-013-0130-4) contains supplementary material, which is available to authorized users.

## Introduction

Early light-induced proteins (ELIPs) were first identified as genes rapidly transcribed after etiolated seedlings were transferred from the dark to the light (Meyer and Kloppstech 1984). ELIPs are members of the light harvesting complex (LHC) superfamily and have three transmembrane domains that traverse the thylakoid membrane. They bind chlorophyll *a* and lutein (Adamska et al. 1999) and are widely distributed throughout the plant kingdom (Adamska 1997). *ELIP* genes are expressed at sparse levels under low light (LL) conditions, but the mRNA quickly becomes abundant in response to high light (HL). ELIP proteins can be detected in the thylakoid membrane within 2 h of HL exposure where they associate with the light harvesting complex of PSII (Heddad and Adamska 2002). ELIP proteins are then degraded soon after the return to LL (Adamska et al. 1993). The rapid responsiveness to HL is conserved throughout the plant kingdom (Heddad and Adamska 2002; Ensminger et al. 2004) and is distinct from the light responsiveness of other LHC family members (Klimmek et al. 2006).

Although sequence and expression patterns are highly conserved, a mechanism of action for ELIPs has not been elucidated by genetic analyses. *Arabidopsis thaliana* contains two *ELIP* genes (*ELIP1*, At3g22840 and *ELIP2*, At4g14690). Overexpression of each *ELIP* gene rescued the photosensitivity of the pleiotropic *chaos* mutant, which lacks cSRP43, and is defective in one of many thylakoid protein insertion pathways. The *chaos* mutant had higher levels of uncoupled chlorophylls, and the rescue by ELIPs suggested a function in sequestering unbound chlorophylls formed during HL (Hutin et al. 2003). However, double *elip1elip2* null mutants displayed no changes in PSII photoinhibition, lipid peroxidation, or qE (nonphotochemical quenching) under HL conditions. The only differences were decreased accumulation of chlorophyll during greening and a decrease in zeaxanthin after HL treatment (Rossini et al. 2006). Overexpression of *ELIP2* resulted in decreased chlorophyll accumulation due to a reduction in chlorophyll synthesis, predominantly at the Mg-chelation step (Tzvetkova-Chevolleau et al. 2007). Both knockout and overexpression of *ELIPs* resulted in decreased chlorophyll levels suggesting a complex relationship between ELIPs and chlorophyll synthesis/accumulation.

Besides HL responsiveness, a Genevestigator perturbations analysis (Hruz et al. 2008) showed significant increases in *ELIP1* and *ELIP2* expression in response to abiotic stresses such as UV-B (Genevestigator ID# AT-00528), cold (AT-00467), heat (AT-00179), drought (AT-00292), hypoxia (AT-00447), and anoxia (AT-00158). Additionally, *ELIPs* were induced in response to *Pseudomonas syringae* systemic infection, a biotic stress (AT-00363). Red (AT-00492), far-red (FR, AT-00109), and blue (AT-00109) light also stimulated *ELIP* expression. Interestingly, the response to red light still occurred in the *phyABCDE* mutant (AT-00601) suggesting the red light response is independent of phytochrome. Despite the inconclusive genetic results described above, these conserved, rapidly activated genes must play an important role in response to light as well as abiotic and biotic stresses.

Photoinhibition within the chloroplast correlates to *ELIP* gene transcription in the nucleus (Heddad et al. 2006), thus the rapid HL-induced expression of *ELIP* genes suggests the operation of chloroplast-nuclear retrograde signaling. Numerous pathways for retrograde signaling have been identified (Kleine and Leister 2013; Kleine et al. 2009), but none explain the rapid induction of *ELIP* genes. Reactive oxygen species are formed under HL, but ELIPs are not induced by superoxide or H_2_O_2_ (Gadjev et al. 2006; op den Camp et al. 2003; Van Aken and Whelan 2012). The carotenoid biosynthesis inhibitor norflurazon activates *ELIP* gene expression, but this activation still occurs in *gun1* and *gun5*, suggesting independence from the tetrapyrrole retrograde signaling pathway (Brusslan and Peterson 2002; Koussevitzky et al. 2007) and ABI4 (Leon et al. 2013). Heme has recently been shown to be a retrograde signaling molecule, and a small dampening of *ELI3* (the Chlamydomonas *ELIP* gene) induction occurs upon bilin feeding in the green algae Chlamydomonas, however *ELI3* induction after a dark to light transition is normal in heme oxygenase mutants that cannot synthesize bilin (Duanmu et al. 2013). Furthermore, *ELIP* expression does not change in distal leaves during systemic acquired acclimation (Rossel et al. 2007). *ELIP2* mRNA levels were higher in *sal1* mutants that cannot produce the retrograde signaling PAP phosphonucleotide, however fold induction in response to HL was normal (Estavillo et al. 2011). It thus appears that *ELIPs* may be induced by a novel retrograde pathway.

Towards understanding *ELIP* retrograde signaling, the pea *ELIP* promoter was studied to identify important *cis* elements. Two well-known light regulatory elements (G-box and GT1) located approximately 120 bp from the start of transcription were implicated in *ELIP* induction when etiolated seedlings were exposed to light. These regions were protected from DNAse digestion by nuclear extracts from both etiolated and light-treated seedlings (Blecken et al. 1994). Both Arabidopsis *ELIP* genes were found to be induced early in response to FR light (Tepperman et al. 2001), and were included in an enumerative screen for promoter elements enriched in early FR-responsive genes (Hudson and Quail 2003). Novel sequences over-represented in light-induced promoters (SORLIP) were identified, with the most highly enriched sequence being SORLIP1 (GCCAC).

In this study, *ELIP* promoter elements that conferred HL responsiveness to a reporter gene were identified using site-directed mutagenesis of full-length promoter-reporter constructs stably integrated into the Arabidopsis genome. Two G-boxes in the *ELIP1* promoter were found to redundantly contribute to HL responsiveness. In addition, a *cis*-region containing double SORLIP1 elements (dSL) was shown to be required for HL responsiveness for both *ELIP1* and *ELIP2* promoters.

## Materials and methods

### Plant material and growth conditions

The *Arabidopsis* *thaliana* (L.) Heynh. (ecotype Columbia or Landsberg *erecta*) plants used in this study were grown in a Percival growth chambers on Sunshine Mix #1 soil (Sun Gro Horticulture Distribution Inc.) under controlled conditions of light, namely LL (≈60 μmol photons m^−2^ s^−1^, 20 h light: 4 h dark) and temperature (23 °C). Light stress was imposed by transferring 21 day-old plants (n > 25 T_2_ seedlings) for 4 h to a Percival E356HO growth chamber (HL ≈ 900 μmol photons m^−2^ s^−1^). HL treatments were performed at the end of the 4 h dark period.

### *ELIP* constructs and *Agrobacterium*-mediated plant transformation

**Fig. 1:**
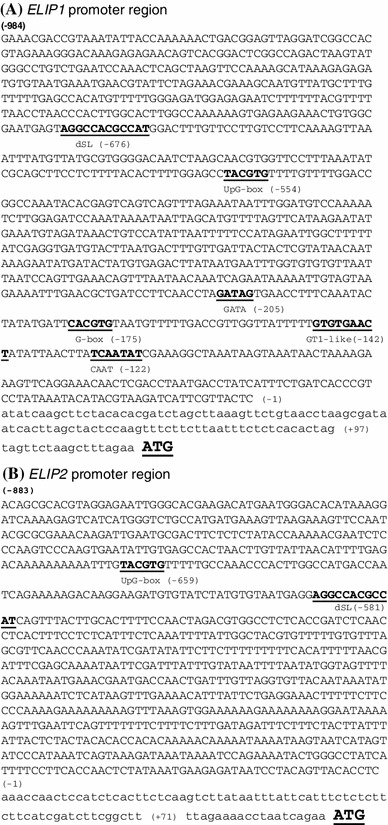
*ELIP1* and *ELIP2* promoter sequences. Sequence characteristics of **a** *ELIP1*- At3g22840 promoter (from −984 to +97 bp) and **b** *ELIP2*-At4g14690 promoter (from −883 to +71 bp). *Upper case letters* correspond to the promoter region and *lower case letters* correspond to the 5′-UTR. The start of translation (ATG) is *underlined* and in *bold*. Below the *cis*-regions that have been subjected to site-directed mutagenesis in this study (in *bold* and *underlined*) are their names and position relative to the start of transcription

The *ELIP1* Wild Type (WT) promoter fragment (1,081 bp = −984 bp to +97 bp of the 5′-UTR) and the *ELIP2* WT promoter fragment (954 bp = −883 bp to +71 bp of the 5′-UTR) were amplified by PCR from *A. thaliana* (ecotype Columbia) genomic DNA (Fig. 1). The primers used for *ELIP1* were: *ELIP1*-forward: 5′-GGAATTCGAAACGACCGTAAATATTACC-3′ and *ELIP1*-reverse: 5′-GCGGATCCCTAGTG TGAGAGAAATTAAG-3′, and for *ELIP2*: *ELIP2*-SalI-Fw: 5′-GGGTCGACACAGCGCACGTAGGAGAATT-3′ and *ELIP2*-Rev: 5′-GCGGATCCAAGCCGAAGATCGATGAAGAAG-3′, introducing an *Eco*RI and *Bam*HI and a *Sal*I and *Bam*HI restriction sites, respectively. These restriction sites were used to clone the promoter fragment into pBluescript II KS plasmid (pKS) (Stratagene, Inc.).

After nucleotide sequence analysis to confirm the integrity of the WT promoter sequence, point mutations were introduced in potential *cis*-acting regions by using the QuickChange^®^ Site Direct Mutagenesis Kit (Stratagene, Inc.). The list of primers used for the site-directed mutagenesis is found in Supplemental Table 1. In the case of the dSL mutated promoter, the first two point mutations in the dSL region were obtained using the primers SL1 (ELIP1) or SL1 (ELIP2). After sequencing and confirming the mutated sequence, the last two point mutations were introduced by using the primers dSL (ELIP1) or dSL (ELIP2) as appropriate. The SL2 (ELIP1) primers were used to create mutations in the second SORLIP1 element, by itself.

All mutated promoter constructs were verified by sequencing, and subcloned into the binary vector pBI101.1 (Clontech Laboratories Inc.) upstream of the *GUS* gene (*uidA)* by using the restriction sites *Sal*I and *Bam*HI. After confirming the integrity of the binary vectors containing the promoter fragment by restriction analysis, these were introduced into *Agrobacterium tumefaciens* (strain GV3101) by electroporation.

Wild type Arabidopsis plants were transformed using the floral dip transformation method (Clough and Bent 1998). T_1_ seeds were collected and then germinated in vitro on medium containing Linsmaier and Skoog salts (Caisson Laboratories Inc.), 20 g L^−1^ sucrose, 7 g L^−1^ agar (Phytablend™, Caisson Laboratories Inc.), 50 mg L^−1^ kanamycin and 50 mg L^−1^ carbenicillin. Antibiotic-resistant seedlings were transferred to soil and T_2_ seeds were collected from individual lines and used directly for experimentation.

The T_2_ plants used in the GUS Assay were also analyzed by PCR and sequencing. Genomic DNA was isolated using DNAzol according to manufacturer’s instructions except that volumes were decreased by 50 % (Invitrogen Inc.). In order to amplify part of the pBI101.1 plasmid and the entire full-length promoter region, the following primers were used: pBI-GUS reverse: 5′-ATGCCCACAGGCCGTCG-3′ and the PBI101.1 forward primer previously listed. This region was then sequenced to confirm that each line had the expected mutations. A partial segment of the GUS gene was also amplified in DNA extracted from T_2_ plants using the following primers: GUS-mid forward: 5′-AAGCCAGACAGAGTG TGATATC-3′ and the GUS-mid reverse: 5′-ATCAATCACCACGATGCCATG-3′. This region was amplified to be certain no deletions of the GUS gene had occurred during the transformation procedure.

### Quantitative fluorometric GUS Assay

T_2_ seeds from each individual line were sown onto moist soil in 2 separate pots (n > 25 seeds per pot) and stratified at 4 °C for 3 days. The pots were then transferred to the growth chamber under the controlled conditions described above. After the LL and HL treatments, three separate samples of 100 mg of leaves were harvested per pot and treatment, containing different T_2_ plants of the same line. Two samples were used to perform the fluorometric Gus assay according to (Jefferson et al. 1987) and the remaining sample was used for RNA extraction. Briefly, leaves were directly ground in 500 μL of extraction buffer. After centrifugation, 50 μl of the supernatant was added to 500 μl of the assay buffer containing the substrate 4-methylumbelliferyl-β-D-glucuronide (MUG) (HACH Inc.) and incubated at 37 °C for 30 min. After 30 min, the reaction was stopped with 900 μl of stop buffer (0.2 M Na_2_CO_3_) and fluorescence due to the product 4-methyl-umbelliferone (4-MU) of the β-glucuronidase activity was measured with the DyNA Quant™ 200 fluorometer (Hoefer Pharmacia Biotech, Inc.). Before measurements, the fluorometer was calibrated with freshly prepared 1 μM 4-MU (Sigma-Aldrich, Inc.) standard and set to 500 relative fluorescence units (RFU). Protein concentration of plant extracts was determined by the Bio-Rad Protein Assay (Bio-Rad, Inc.), RFU values were normalized to the protein concentration in individual samples and Gus Activity was expressed as nmoles min^−1^ mg^−1^. Data were expressed as HL/LL fold induction.

### Real time qPCR

RNA was isolated using Trizol reagent (Invitrogen Inc.), and cDNA was synthesized using random hexamers (Operon Biotechnologies Inc.) and MMLV reverse transcriptase (New England Biolabs Inc.). cDNA was diluted 1:3 prior to real-time PCR. Real time PCR amplification was performed in an MX3000P real-time PCR machine (Stratagene, Inc.) using 2× SYBR Green mix (AB Gene Inc.) in a total volume of 12 μL. PCR reactions all used a 61 °C annealing temperature, and dissociation curves were done to check for primer-dimers. The primers were as follows: ACT2-F: 5′-GGCGACTTGACAGAGAAGAA; ACT2-R: 5′-TGGAAAGAAAGAGCGGAAGA; Gus-Fw1: GAACTGAACTGGCAGACTATCCC; Gus-Rev1: 5′-TCGGCGTGGTGTAGAGCATTAC; Elip1-qRT-Fw: 5′-AAGGTGGGACACTCGTCTAAG; Elip1-qRT-Rv: 5′-GTGTTTTTAACCCGAAGTTTC; Elip2-qRT-Fw2: 5′-CCACAAATGCCACAGTCTC, Elip2-qRT-Rv2: 5′-CTCCAAACTTCGTACTCACC.

### Statistical analysis

The non-parametric Mann–Whitney test (Mann and Whitney 1947) was used to determine significant differences in pairwise comparisons for GUS activity and mRNA analyses.

## Results

### Identification of *ELIP* promoter elements conferring HL induction

**Fig. 2:**
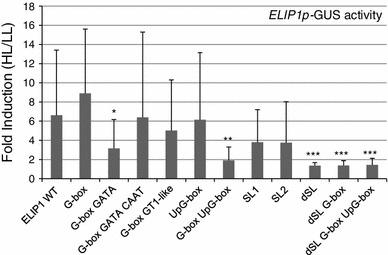
GUS activity in ELIP1p-GUS transgenic lines. A 984 bp region of *ELIP1p* was fused to the GUS reporter gene (ELIP1 WT). Site-directed mutants of the 984 bp region were generated and also fused to the GUS reporter gene. Transgenic lines were generated and tested for GUS activity in LL and after 4 h of HL to produce the HL/LL fold induction. 20–25 transgenic lines were analyzed for each construct and error bars indicate the interquartile range (IQR). The location and sequence of promoter elements are shown in Fig. 1 while the nucleotide changes for each element are shown in Table 1. The SL1 and SL2 mutants change one of the two SORLIP1 elements in the dSL region. Pairwise comparisons between ELIP1 WT and each mutant construct were evaluated for statistical significance using the Mann–Whitney test. *p value 0.0500–0.0100, **p value 0.009–0.001, ***p value 0.0009 or below, but greater than 0

To define promoter elements that play a role in HL-induced *ELIP1* expression, site-directed mutagenesis was performed on the *ELIP1* promoter (*ELIP1p*) using a region that extended 984 bp upstream from the start of transcription and included 97 bp of the 115 bp 5′-UTR (Fig. 1). This region was cloned upstream of the GUS reporter gene and conferred a strong induction of GUS activity after 4 h of HL treatment (ELIP1 WT, Fig. 2). *ELIP1* mRNA showed maximal induction after 3 h of HL exposure, and a 4 h HL exposure was utilized in all experiments to permit accumulation of GUS protein (Supplemental Figure 1). Numerous well-defined light regulatory elements (LREs) (Arguello-Astorga and Herera-Estrella 1998; Higo et al. 1999; Kuhlemeier et al. 1987) are located in the *ELIP1* promoter: CAAT at -122 (relative to the start of transcription), GT1-like at -142, G-box at -175, GATA at -205 and upstream G-box (UpG-box) at -554. Full-length promoter regions with the LRE-element nucleotide changes shown in Table 1 were constructed as single, double and triple mutants, introduced into *Arabidopsis* via *Agrobacterium* floral dip transformation, and individual transgenic lines were selected and tested for HL induction of GUS activity in the T_2_ generation. Figure 2 shows the HL/LL fold induction for each construct (n = 20–25 transgenic lines). Most LREs did not significantly affect GUS HL/LL induction, however double mutations in both the G-box and UpG-box significantly (*p* = 0.0010) decreased GUS HL/LL induction in comparison to *ELIP1* WT, suggesting the additive importance of these two elements. A small and slightly significant difference (*p* = 0.0243) was observed upon mutation of both the G-box and GATA elements, however a triple mutant (G-box, GATA, and CAAT) was not significantly different than *ELIP1* WT. These findings suggest the GATA box has a small positive effect, while the CAAT box has a small negative effect on *ELIP1* promoter activity.

**Table 1:** *ELIP* promoter motifs and site-directed mutations

Promoter Motif	Location	Wild-type	Site-directed mutant
CAAT	−122	TCAATA	TCCCTA
GT1-like	−142	GTGTGAACT	GCGCGAACT
G-box	−175	CACGTG	CCCGGG
GATA	−205	AGATAG	ATCTAG
Upstream G-box	−554	TACGTG	TCCGGG
*ELIP1* dSL	−676	AGGCCACGCCAT	AGACCCCACTAT
SL1	−676	AGGCCACGCCAT	AGACCCCGCCAT
SL2	−676	AGGCCACGCCAT	AGGCCACACTAT
*ELIP2* dSL	−581	AGGCCACGCCAT	AGACCCCACTAT

Wild type and site-directed mutant promoter motifs are shown relative to the start of transcription. All site-directed mutants were made in the context of a full-length promoter (984 bp for *ELIP1* and 883 bp for *ELIP2*). dSL and dSLm from *ELIP1* and *ELIP2* are identical, but their location differs by 95 bp

In order to find additional elements, *ELIP1p* and *ELIP2p* were analyzed using the Arabidopsis Promoter Element Discovery Tools (http://stan.cropsci.uiuc.edu/tools.php), and the SORLIP1 element (GCCAC) was found to be over-represented (*p* = 1.80e−04). Interestingly, a near perfect duplication of the SORLIP1 element (AGGCCAC GCCAT) within a completely conserved 12 bp region was found at −676 of *ELIP1* and −581 of *ELIP2*. This 12 bp region is only found in the −1,000 region of one other expressed Arabidopsis gene, At2g38530, which encodes a stress-induced lipid transfer protein. This element was named double SORLIP1 (dSL), and subjected to site-directed mutagenesis of either one or both SORLIP1 elements (Table 1). *ELIP1p* with site-directed mutations singly disrupting the dSL SORLIP1 elements (SL1 and SL2) did not display significantly reduced HL/LL induction of GUS activity, however *ELIP1p* with both dSL SORLIP1 elements disrupted did show a significant reduction in HL/LL induction of GUS activity (*p* < 0.00009, Fig. 2). dSL mutants in combination with one or both G-box elements did not further decrease HL/LL induction of GUS activity suggesting that G-boxes and dSL are not additive.

Mutations in promoter regions can lead to complete inactivation. To demonstrate this had not occurred for dSL mutants, LL GUS activity levels were plotted in relation to HL/LL GUS fold induction in Supplemental Figure 2. Although the highest LL activity levels were observed for a subset of *ELIP1p* WT transgenic lines, many lines with greater than 1 nmole min^−1^ mg^−1^ of GUS activity were observed in the *ELIP1p* dSL mutant transgenic lines.

**Fig. 3:**
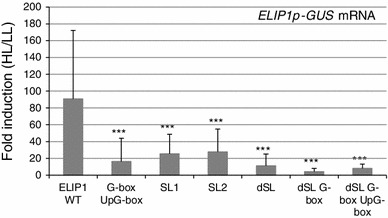
*GUS* mRNA levels in ELIP1p-GUS transgenic lines. ELIP1p-GUS lines were tested for RNA expression by real-time qPCR harvested from the same tissue used for GUS activity measurements. Both *GUS* and native *ELIP1* mRNA levels were quantified and samples that displayed a less than twofold induction of native *ELIP1* were removed from the analysis. *GUS* mRNA levels with a significantly lower HL/LL fold induction than ELIP1p WT are shown in *white bars*. The number of transgenic lines analyzed varied among constructs: ELIP1 WT (15), G-box-UpG-box (14), SL1 (8), SL2 (20), dSL (26), dSL-G-box (19), dSL-G-box-UpG-box (16). *Error bars* represent IQR. Statistical analysis and *p* values are as indicated for Fig. 2

For a subset of constructs, tissue grown for GUS activity assays was also harvested for RNA extraction, and native *ELIP1* and *GUS* mRNA were quantified by real-time qPCR using *ACT2* as a reference (Livak and Schmittgen 2001). Expression of the native *ELIP1* served as an internal control for HL induction and any samples that had native *ELIP1* induction levels less than twofold were removed from the statistical analysis. Figure 3 shows the HL/LL fold induction for *ELIP1p*-*GUS* mRNA, and significant differences compared to WT were observed for the G-box UpG-box double mutant (*p* = 0.0002) as well as each single SORLIP1(*p* = 0.0009 for SL1 and 0.0006 for SL2). The dSL mutant and the dSL mutant combined with G-box promoter mutants were highly significantly different than WT (*p* < 0.00009 in all cases). Overall, the mRNA data show that the dSL is required for HL induction and that each SORLIP1 element within the dSL contributes to HL/LL fold induction along with the G-box and the UpG-box.

**Fig. 4:**
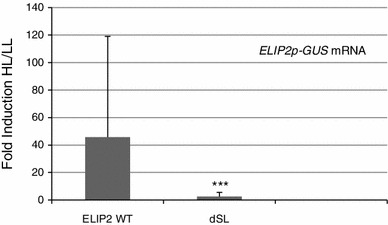
The dSL element of *ELIP2* is required for HL induction. ELIP2 WT (n = 14) and dSL mutant (n = 15) transgenic lines were tested for native *ELIP2* and *GUS* mRNA expression by real-time qPCR. Samples that displayed a less than twofold induction of native *ELIP2* were removed from the analysis. *Error bars* represent IQR. Statistical analysis and *p* values are as indicated for Fig. 2

If the dSL element is important for HL induction, it would be expected to play a similar role in the HL-inducible *ELIP2* promoter, where it is present in a similar position (Fig. 1). Site directed mutagenesis was carried out in the context of a full-length *ELIP2p* (−883 to +71 of an 80 bp 5′-UTR), and both *ELIP2p* WT and *ELIP2p* dSL mutant promoters were cloned adjacent to the GUS reporter. Transgenic lines, were treated with HL and *ELIP2p*-*GUS* and native *ELIP2* mRNA were quantified and any samples that had native *ELIP2* induction levels less than twofold were removed from the statistical analysis. A significant decrease in HL/LL fold induction of *GUS* mRNA for the *ELIP2p* dSL mutant compared to the *ELIP2p* WT promoter construct was observed (*p* < 0.00009). These data show that the dSL element is required for *ELIP2p* to confer HL/LL induction on a reporter construct (Fig. 4).

To view the variability of the transgenic lines, *GUS* mRNA induction was plotted against native *ELIP1* mRNA levels in Supplemental Figure 3a. The bulk of *ELIP1p* dSL mutant lines were clustered towards the y-axis, while many *ELIP1p* WT lines showed high HL/LL induction of *GUS* mRNA. A similar analysis with the *ELIP2p* constructs is shown in Supplemental Figure 3b. The high variability is most likely due to random integration of different copies of T-DNA constructs into more active and less active chromatin regions (Butaye et al. 2005).

### SORLIP1 elements in *ELIP* promoter regions throughout the plant kingdom

**Fig. 5:**
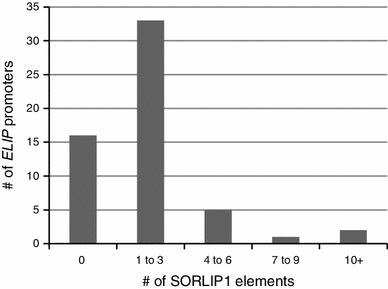
SORLIP1 elements in *ELIP* promoters throughout the plant kingdom. 37 *ELIP* promoters throughout the plant kingdom were identified and 1,500 bp upstream from the start of translation was scanned for SORLIP1 elements (GCCAC). The number of promoters (y-axis) with different numbers of SORLIP1 elements (x-axis) is shown. SORLIP1 elements were enriched 1.5 fold in *ELIP* promoter regions compared to random occurrence

**Fig. 6:**
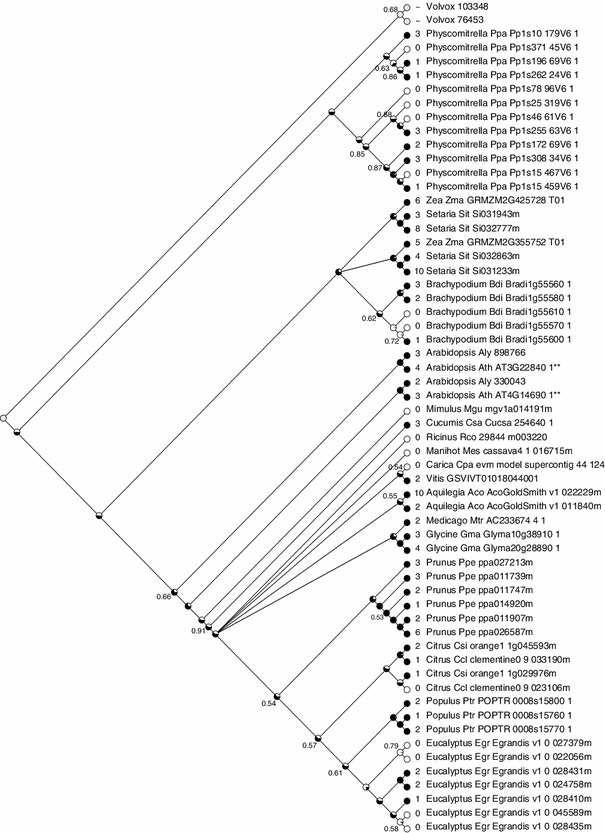
SORLIP1 element gain/loss in *ELIP* promoter regions throughout the plant kingdom. *ELIP* promoters were scanned for SORLIP1 elements (GCCAC) and a phylogenetic tree was generated using Mesquite. *Black* signifies presence of at least one SORLIP1 element. The probability of a common ancestor having one or more SORLIP1 elements is shown by the proportion of *black* in common ancestor *circles*. The *numbers* represent maximum likelihood values supporting the ancestral proportions

To determine the distribution of SORLIP1 elements in *ELIP* promoter regions, 57 *ELIP* gene promoters from non-vascular as well as vascular plants were scanned for GCCAC sequences located 1,500 bp upstream from the start of translation. Most *ELIP* promoters contained 1–3 SORLIP1 elements (Fig. 5), however only the Arabidopsis genes contained the dSL element. When the frequency of SORLIP1 elements was compared to the random occurrence of any 5 bp region, SORLIP1 elements were found to be 1.5 fold enriched. This number is likely an underestimate since promoter regions tend to be AT-rich (Morey et al. 2011). *ELIP* genes were placed into a phylogenetic tree using Mesquite which displays the presence/absence of a trait (Maddison and Maddison 2011). The trait tracked was the presence of one or more SORLIP1 elements (Fig. 6). *ELIP* promoters with SORLIP1 elements were widely distributed among moss, monocots and dicots. For species with many *ELIP* paralogs, such as *Eucalyptus grandis*, specific clades had lost SORLIP1 elements suggesting functional diversification and not just random gain/loss, however gain/loss was more widely distributed for the *Physcomitrella* *patens* *ELIP* paralogs.

## Discussion

A transgenic promoter-reporter system was used to define elements within *ELIP1p* that are essential for responsiveness to HL. Defining these elements provides endpoint information on the retrograde signaling mechanism that regulates *ELIP* gene expression in response to HL. Large numbers of transgenic lines (14–27) were used to overcome the position effects that lead to high variability in reporter gene expression. Many classic LREs (GATA and CAAT) were found to only marginally contribute to HL activation by *ELIP1p*. However, two G-boxes, located at −175 and −554, redundantly increased HL/LL fold induction of GUS mRNA and activity. G-boxes have been implicated in HL activation previously (Blecken et al. 1994), however the extent of their contribution is unclear since microarray experiments show strong HL-induction of both *ELIP1* and *ELIP2* in the *hy5* mutant, which encodes a bZIP transcription factor that binds to G-boxes (AT-00246) (Chattopadhyay et al. 1998). It is possible that other G-box binding TFs are involved in HL induction. The previous analysis of the pea *ELIP* promoter suggested that the GT1 element may be important (Blecken et al. 1994), but our study did not support a role for the *ELIP1* GT1-like element. The pea study used different conditions to activate *ELIP* expression (etiolated seedlings transferred to low light as opposed to mature leaves exposed to HL) and utilized promoter deletions instead of site-directed mutagenesis of the full-length promoter.

GUS activity and mRNA levels had a significantly reduced response to HL when the dSL element was subjected to site-directed mutagenesis. The dSL element consists of two adjacent SORLIP1 elements, with the second one having a single nucleotide substitution, in a 12 bp region conserved between *ELIP1p* and *ELIP2p*. Mutagenesis of each SORLIP1 element resulted in lower HL induction of *GUS* mRNA, however a significant difference was not observed for GUS activity. Quantification of mRNA is a more direct measurement of promoter activity, and is more likely to reflect the importance of promoter elements. The *ELIP1* dSL mutant promoters consistently showed a reduction in reporter induction after HL exposure, which was not reversed or strengthened by mutation in other elements. In addition, the dSL element was required for HL induction in *ELIP2p*. Taken together, our transgenic promoter-reporter analysis identified SORLIP1 elements to be essential for HL induction conferred on reporters for both Arabidopsis *ELIP* promoters. Interestingly, the minimal region that could confer light responsiveness (−228 to −74) in the earlier pea study did contain one SORLIP1 element at −110 (Kolanus et al. 1987), thus the pea study could also support a role of SORLIP1 elements in *ELIP* expression.

*ELIP* promoters throughout the plant kingdom were scanned for SORLIP1 and dSL elements. dSL elements were only found in the *A. thaliana* *ELIP* promoters, but SORLIP1 elements were distributed widely and a low level of enrichment (1.5×) was estimated based on the random occurrence of the five bp sequence. Two *ELIP* promoters had 10 SORLIP1 elements (*Setaria* Si031233 and *Aquilegia* 022229) while 28 % had no SORLIP1 sequences. A phylogenetic tree of the *ELIP* sequences showed a broad distribution for the presence of at least one SORLIP1 element. Three *ELIP* gene clades were found in *Eucalyptus grandis*, but only one clade contained *ELIP* promoters with SORLIP1 elements. In *Brachypodium distachyon*, one clade had 2–3 SORLIP1 elements while the other had 0–1 SORLIP1 elements. It will be interesting to determine if HL induction levels for Eucalyptus and *Brachypodium* *ELIP* paralogs correlate with the presence/absence of SORLIP1 elements.

The dSL element plays a role in HL induction, but *ELIP* genes have been shown to be regulated by other abiotic and biotic stresses (Hruz et al. 2008). To determine if the dSL element is important in modulating an increase in expression in response to these stresses, WT and dSL mutant transgenic lines will need to be exposed to these stresses and *GUS* and *ELIP* mRNA levels will need to be quantified to determine if there are significant differences in *GUS* expression.

A yeast one-hybrid screen was performed to identify dSL interacting partners, and the C-terminal region of Lhca2 was selected at a high frequency (11 of 30 in-frame clones, Supplemental Figure 4a). Lhca2-encoding clones displayed strong activation with the dSL bait, but no activation for the dSL mutated (dSLm) bait (Supplemental Figure 4b). This specificity was surprising since Lhca2 is a light harvesting complex protein associated with PSI and localized to the chloroplast thylakoid membrane. The C-terminal region of Lhca2 has no cryptic DNA binding domains or a nuclear localization sequence (Rost et al. 2003). The high abundance of *LHCB* and *LHCA* mRNAs would result in a high abundance of LHC-encoding cDNAs, however if the *LHCA2* clones were random selection artifacts, many other *LHCB* and *LHCA* cDNAs would have been selected as well: not just one region of one LHC-encoding cDNA. A genetic analysis (*ELIP1* and *ELIP2* mRNA induction after HL treatment in an *lhca2* T-DNA insertion mutant (Alboresi et al. 2009)) did not support a role for Lhca2 in HL induction of *ELIP* mRNA, and thus the specific activation of the dSL bait by the C-terminal region of Lhca2 observed in yeast does not appear to extend to Arabidopsis.

In conclusion, the double SORLIP1 element has been shown to be required for HL induction of *ELIP1* and *ELIP2* promoters in *A. thaliana* using transgenic lines with promoter-reporter constructs. In addition, two G-box elements redundantly contributed to the HL induction of the *ELIP* genes. *ELIP* gene promoters throughout the plant kingdom display a small level of enrichment for SORLIP1 elements.

## Electronic supplementary material

Below is the link to the electronic supplementary material.

Supplementary material 1 (DOCX 1039 kb)
