# Supplementary material for: A double SORLIP1 element is required for high light induction of ELIP genes in Arabidopsis thaliana
Source: Plant Mol Biol. 2013 Sep 27;84(3):259–67. doi: 10.1007/s11103-013-0130-4 (PMC3889287; doi:10.1007/s11103-013-0130-4)
Supplement: Supplementary file 1 — Supplementary material 1 (DOCX 1039 kb) [file 11103_2013_130_MOESM1_ESM.docx]

**Supplemental Table 1** Primers used for site directed mutagenesis. The *cis*-regions are underlined and mutated nucleotides within the *cis*-region are in bold.

| *cis* regions | Primers used for site directed mutagenesis |
| --- | --- |
| CAAT (ELIP1) | CAAT fw: 5’-GTGTGAACTATATTAACTTATC**CC**TATCGAAAGGCTAAAT AAG-3’  CAAT rv: 5’-CTTATTTAGCCTTTCGATA**GG**GATAAGTTAATATAGTT CACAC-3’ |
| GT1-Like (ELIP1) | GT1-like fw: 5’-CCGTTGGTTATTTTG**C**G**C**GAACTATATTAACT TATCAATATC-3’  GT1-like rv5’-GATATTGATAAGTTAATATAGTTC**G**C**G**CAAA ATAACCAACGG-3 |
| G-box (ELIP1) | G-box fw 5’-CCTTTCAAATACTATA TGATTC**C**CG**G**GT AATGTTTTTGACCG TTGG-3’  G-box rv: 5’-CCAACGGTCAAAAACATTAC**C**CG**G**GAATCATATAGTATTTGAAAGG-3’ |
| GATA (ELIP1) | GATA fw: 5’-GAACGCTGATCCTTCAACCTA**TC**TAGTGAACCTTTCAAATAC-3’  GATA rv: 5’-GTATTTGAAAGGTTCACTA**GA**TAGGTTGAAGGATCAGCGTTC-3’ |
| UpG-box (ELIP1) | UpG-box fw: 5’-CCTCTTTTACACTTTTGGAGCCT**C**CG**G**GTTTTGTTTTGGACCG-3’  UpG-box rv: 5’-CGGTCCAAAACAAAAC**C**CG**G**AGGCTCCAAAAGTGTAAAAGAGG-3’ |
| SL1 (ELIP1) | First Two fw 5’- CGAATGAGTAG**A**CC**C**CGCCATGGACTTTGTTCCTTGTCC-3’  First Two rv: 5’-GGACAAGGAACAAAGTCCATGGCG**G**GG**T**CTACTCATTCG-3’ |
| SL2 (ELIP1) | Last Two fw: 5’-CGAATGAG TAGGCCAC**A**C**T**ATGGACTTTGTTCCTTGTCC-3’  Last Two rv: 5’-GGACAAGGAACAAAGTCCAT**A**G**T**GTGGCCTACTCATTCG-3’ |
| dSL (ELIP1) | 12 bp fw: 5’-CGAATGAGTAG**A**CC**C**C**A**C**T**ATGGACTTTGTTCCTTGTC C-3’  12 bp rv: 5’-GGAC AAGGAACAAAGTCCAT**A**G**T**G**G**GG**T**CTACTCATTC G-3’ |
| SL1 (ELIP2) | Elip2-12bp-1Fw: 5’-TAATGAGGAG**A**CC**C**CGCCATCAGTTTACTTGCACTTTTCC  Elip2-12bp-1Rev: 5’-GGAAAAGTGCAAGTAAACTGATGGCG**G**GG**T**CTCCTCATTA-3’ |
| dSL (ELIP2) | Elip2-12bp-2Fw: 5’-TAATGAGGAG**A**CC**C**C**A**C**T**ATCAGTTTACTTGCACTTTTCC-3’  Elip2-12bp-2Rev: 5’-GGAAAAGTGCAAGTAAACTGAT**A**G**T**G**G**GG**T**CTCCTCATTA-3’ |

**Supplemental Fig. 1** Relative expression of *ELIP1* after exposure to LL (60 μmoles photons m^-2^ sec^-1^) or HL (900 μmoles photons m^-2^ sec^-1^) for the indicated number of hours. Dark expression levels are set to 1. Plants were grown in a 20h light: 4h dark diurnal cycle and exposed to light at the end of the 4h dark period.

**Supplemental Fig. 2** ELIP1p individual transgenic lines GUS activity data. Data are shown for individual transgenic lines for the WT *ELIP1* promoter (ELIP1p, n = 27) and the dSLm promoter (ELIP1p dSLm, n = 26). For each line, GUS activity is shown with HL/LL fold induction value on the y-axis and the LL expression level (nmoles min^-1^ mg^-1^) on the x-axis. For the ELIP1p WT promoter, some individual lines had high LL activity, but this did not associate with high levels of HL/LL fold induction. Lines with similar LL GUS activity can be compared, and the ELIP1p WT promoters show much greater HL/LL fold induction.

**Supplemental Fig. 3** ELIPp Individual Transgenic Line mRNA Data. ELIP1p-GUS (top panel) and ELIP2p-GUS (bottom panel) lines were grown under LL and transferred to HL for 4 h. Native *ELIP* and *ELIPp-GUS* mRNA levels were quantified by real-time qPCR and displayed for each line in the scatter plot. Induction of the corresponding native *ELIP* mRNA was observed in all transgenic lines (y-axis), however *GUS* mRNA (x-axis) was increased with ELIPp but not with ELIPp dSLm for both *ELIP1p* and *ELIP2p*.

**Supplemental Fig. 4**  Yeast One-Hybrid Screening. a. The dSL element was used as bait in the yeast one-hybrid screen, and of 40 plasmids isolated from aureobasidin^r^ colonies and sequenced, 11 encoded the C-terminal region of Lhca2. Of the11 regions encoded by the cDNAs, three began at L176, four began at T177, two began at G178, and one each started with E184 and R186. The downward arrow indicates the chloroplast import cleavage site. b. cDNA encoding 80 aa C-terminal region of Lhca2 (beginning at G178) was then tested for specificity with dSL and dSLm baits. Although colonies were plentiful with the dSL bait, no aureobasidin^r^ colonies were observed with the dSLm bait. A typical false positive cDNA encoding HTB4 activated both the dSL and dSLm baits. The concentration of aureobasidin was 100 ng/μL.
